# Supplementary material for: Predicting transdermal fentanyl delivery using physics-based simulations for tailored therapy based on the age
Source: Drug Deliv. 2022 Mar 23;29(1):950–69. doi: 10.1080/10717544.2022.2050846 (PMC8956318; doi:10.1080/10717544.2022.2050846)
Supplement: Supplemental Material [file IDRD_A_2050846_SM4037.docx]

# SUPPLEMENTARY MATERIAL

**S.1. Sensitivity Analysis over the parameters**

The results of the sensitivity analysis over model input parameters are demonstrated in Figure s1. In Figure s1/a, the sensitivity index of the fentanyl flux out of the dermis to input parameters for the drug uptake model is shown. These input parameters are partition and diffusion coefficient in the patch and skin layers and the thickness of the skin layers thickness. Based on this result, the fentanyl flux was most sensitive to epidermis thickness and diffusion in the epidermis. Based on the sensitivity index, the flux is not so sensitive to equivalent dermis thickness; however, this result is for a 1% change in the value of the equivalent dermis thickness. It should be noted that the changes in equivalent dermis thickness could be notable. Its thickness can change from not considering the dermis layer (assuming drug uptake at the interface of the epidermis and dermis) to considering the whole thickness of the dermis (a few millimeters). Therefore, despite the obtained low sensitivity index for dermis thickness, dermis thickness could play an important role in the outcome of the model.

In Figure s1/b, the sensitivity of the average plasma concentration to the volume of the compartments, inter-compartmental clearance, renal and hepatic clearance, and the fraction of unbound drug is shown. The average concentration of fentanyl in the plasma is largely sensitive to the fraction of unbound drug. Between the blood and other compartments, and the only unbounded drug can be transferred; therefore, this parameter has a huge impact on fentanyl concentration in plasma. Another important parameter in the PK model is the blood flow to the gastrointestinal compartment. Unlike other compartments, in which outgoing flow gets back to the central compartment, for the gastrointestinal compartment, the outgoing flow goes to the liver. Therefore, the blood flow of the gastrointestinal compartment will affect the amount of metabolized the drug. The sensitivity index of the average VAS pain score to the Half maximal effective concentration and Hill coefficient in Figure s1/c shows that the concentration of half-maximum effect has an important role in the resulting effect.


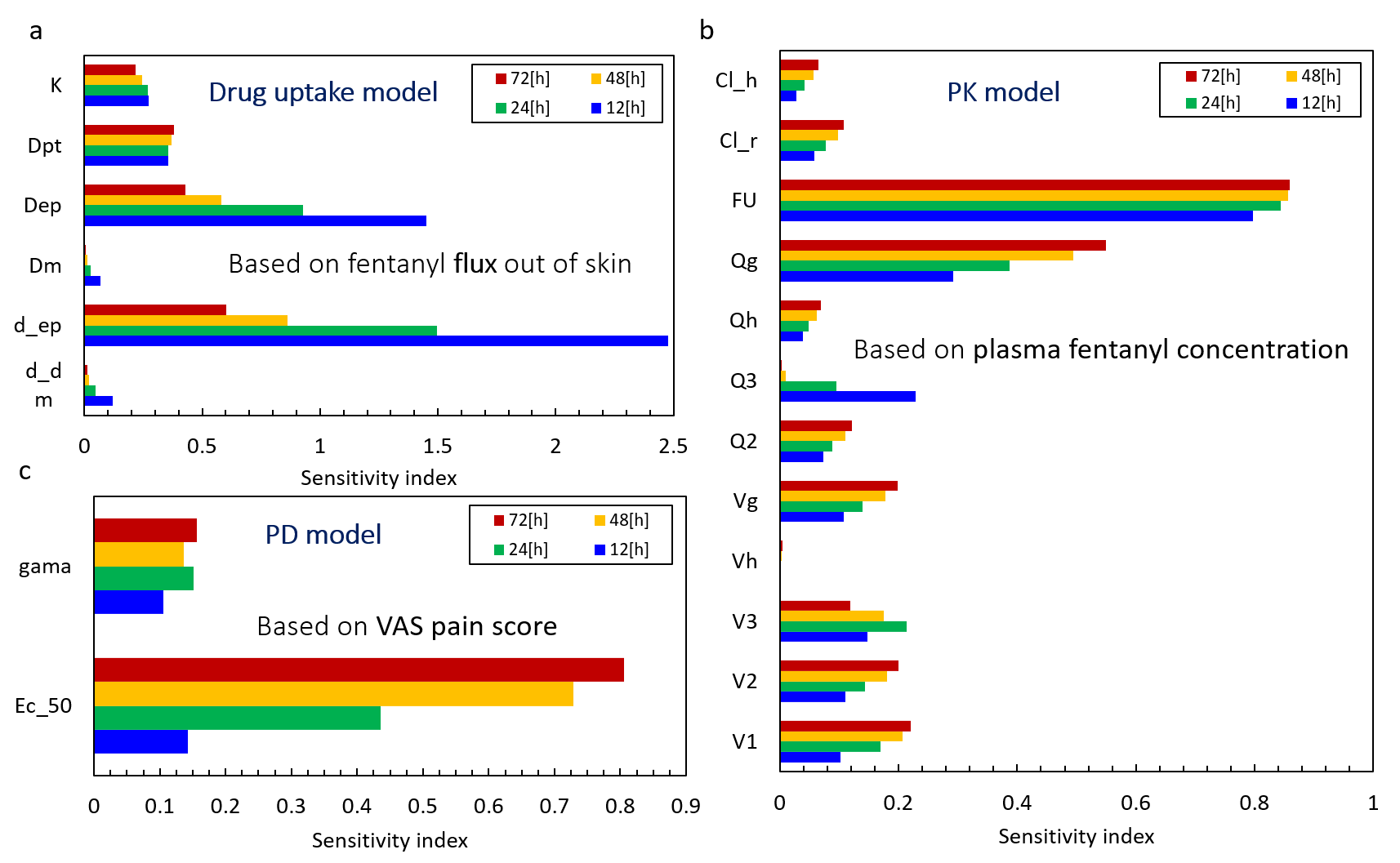


Figure s1- Sensitivity analysis results, a: sensitivity index of fentanyl flux out of dermis to drug uptake model parameters. b: sensitivity index of plasma fentanyl concentration to PK model parameters. c: sensitivity index of VAS pain score to the PD model parameter. The analysis was done over 72 hours of therapy for the virtual patient at the age of 20 years with Duragesic® fentanyl patch with the nominal flux of 75 µg h^-1^
